# Body mass index trajectories from 2 to 18 years – exploring differences between European cohorts

**DOI:** 10.1111/ijpo.12115

**Published:** 2016-02-26

**Authors:** L. Graversen, L. D. Howe, T. I. A. Sørensen, U. Sovio, L. Hohwü, K. Tilling, J. Laitinen, A. Taanila, A. Pouta, M‐R. Järvelin, C. Obel

**Affiliations:** ^1^ Section for General Medical Practice, Department of Public HealthAarhus UniversityAarhusDenmark; ^2^ MRC Integrative Epidemiology UnitUniversity of BristolBristolUK; ^3^ School of Social and Community MedicineUniversity of BristolBristolUK; ^4^ Institute of Preventive MedicineBispebjerg and Frederiksberg HospitalsCopenhagenDenmark; ^5^ Novo Nordisk Foundation Center for Basic Metabolic Research, Faculty of Health and Medical SciencesUniversity of CopenhagenCopenhagenDenmark; ^6^ Department of Obstetrics and GynaecologyUniversity of CambridgeCambridgeUK; ^7^ Department of Epidemiology and BiostatisticsImperial CollegeLondonUK; ^8^ Finnish Institute of Occupational HealthHelsinkiFinland; ^9^ Institute of Health SciencesUniversity of OuluOuluFinland; ^10^ Unit of Primary CareUniversity Hospital of OuluOuluFinland; ^11^ National Institute of Health and WelfareOuluFinland; ^12^ Department of Obstetrics and GynecologyUniversity of Oulu and Oulu University HospitalOuluFinland; ^13^ Biocenter OuluUniversity of OuluOuluFinland; ^14^ Center for Life Course EpidemiologyUniversity of OuluOuluFinland

**Keywords:** ALSPAC, BMI, child, cohort studies

## Abstract

**Background:**

In recent decades, there has been an increase in the prevalence of childhood overweight in most high‐income countries. Within northern Europe, prevalence tends to be higher in the UK compared with the Scandinavian countries. We aimed to study differences in body mass index (BMI) trajectories between large cohorts of children from UK and Scandinavian populations.

**Methods:**

We compared BMI trajectories in participants from the English Avon Longitudinal Study of Parents and Children born in 1991–1993 (ALSPAC) (N = 6517), the Northern Finland Birth Cohorts born in 1966 (NFBC1966) (N = 3321) and 1986 (NFBC1986) (N = 4764), and the Danish Aarhus Birth Cohort born in 1990–1992 (ABC) (N = 1920). We used multilevel models to estimate BMI trajectories from 2 to 18 years. We explored whether cohort differences were explained by maternal BMI, height, education or smoking during pregnancy and whether differences were attributable to changes in the degree of skew in the BMI distribution.

**Results:**

Differences in mean BMI between the cohorts were small but emerged early and persisted in most cases across childhood. Girls in ALSPAC had a higher BMI than all other cohorts throughout childhood, e.g. compared with the NFBC1986 BMI was 2.2–3.5% higher. For boys, the difference emerging over time (comparing the two NFBC's) exceeded the differences across populations (comparing NFBC1986, ABC and ALSPAC). BMI distribution demonstrated increasing right skew with age.

**Conclusion:**

Population‐level differences between cohorts were small, tended to emerge very early, persisted across childhood, and demonstrated an increase in the right‐hand tail of the BMI distribution.

## Introduction

Childhood obesity has become a worldwide public health challenge, as the prevalence of childhood overweight has increased over time in all countries 1, 2. There are, however, considerable international 3, 4 and regional 2, 5 differences in the prevalence of childhood overweight. For example, a prevalence of overweight including obesity among 10–16 years old in 2001 and 2002 of 11.4% in Denmark, 14.3% in Finland and 18.4% in England has been reported 5. Most of our knowledge in this field is based on cross‐sectional data 1, 2, 3, 4, 5. Little is known about how early in life these differences in body mass index (BMI) emerge. Our aim was to compare BMI development in four large birth cohorts; two from Finland born 20 years apart, one from the United Kingdom and one from Denmark.

## Cohorts and methods

We used data from the Northern Finland Birth Cohorts born in 1966 (NFBC1966) and 1986 (NFBC1986), the Danish Aarhus Birth Cohort born in 1990–92 (ABC) and the English Avon Longitudinal Study of Parents and Children born in 1991–1993 (ALSPAC).

For all four cohorts, participants were recruited during pregnancy and were asked to fill in structured questionnaires, including information on maternal smoking, height and weight. Data on pregnancy, birth and childhood growth were collected prospectively. In the NFBC's, data on postnatal growth up till adolescence were obtained from the routine healthcare records on children participating in a follow‐up visit at the age of 31 years in NFBC1966 and 16 years in NFBC1986. In ABC, health nurses and general practitioners were contacted to obtain postnatal growth data from routine healthcare records. In ALSPAC, the follow‐up included parent and child completed questionnaires, data extraction from routine healthcare records and research clinic attendance. Only singletons born after 37 weeks of gestation were included in the present study. All four study populations comprised above 95% White European.

### Northern Finland Birth Cohorts born in 1966

Northern Finland Birth Cohorts born in 1966 consist of 96.3% of all children who were due to be born in the provinces of Oulu and Lapland in Northern Finland in 1966, and 12 058 live‐born children entered the study 6, 7. Subjects living in the original target area or in the capital area were invited to a clinical examination at the age of 31 years and 71% took part. Growth data were available for 4036 term born singletons. Signed, informed consent and written permission to use their data for scientific research was obtained from the study participants at the age of 31. The study website contains details of the data (http://www.oulu.fi/nfbc). The University of Oulu Ethics Committee approved the study.

### Northern Finland Birth Cohorts born in 1986

Northern Finland Birth Cohorts born in 1986 consist of 99% of all children who were due to be born in the provinces of Oulu and Lapland in Northern Finland between 1 July 1985 and 30 June 1986, and 9432 live‐born children entered the study. Subjects living in the original target area or in the capital area were invited to a clinical examination at the age of 16 years, and 74% took part. Growth data were available for 5305 term born singletons. At the age of 16 years, the adolescents and their parents gave informed consent and written permission to use their data for scientific research. The study website contains details of the data (http://www.oulu.fi/nfbc). The University of Oulu Ethics Committee approved the study.

### Danish Aarhus Birth Cohort

Danish Aarhus Birth Cohort consists of 8719 Danish‐speaking women with a singleton pregnancy seen for their first antenatal visit between 1 August 1989 and 30 September 1991 at the Department of Obstetrics and Gynaecology, Aarhus University Hospital 8. In 2001, children in the cohort were invited to participate in a follow‐up study. A total of 7953 mother–child pairs received a child health questionnaire and were asked for a signed consent to collect historical data of the child, and permission was given by 6504 parents. Growth data were available for 4810 term born singletons. The study was approved by the Danish Central Ethical Committee.

### English Avon Longitudinal Study of Parents and Children

English Avon Longitudinal Study of Parents and Children consists of the offspring of pregnant women resident in one of three Bristol based health districts with an expected delivery date between 1 April 1991 and 31 December 1992 9. A total of 14 062 live‐born children entered the study. Growth data were available for 13 491 term born singletons. The study website contains details of all the data that is available through a fully searchable data dictionary (http://www.bris.ac.uk/alspac/researchers/data‐access/data‐dictionary). Ethical approval for the study was obtained from the ALSPAC Ethics and Law Committee and the Local Research Ethics Committees.

### Statistical analyses

We used measurements of height and weight from 2 to 18 years to calculate BMI (kg/m^2^). We included children with at least one measurement between 2 and 5 years, one measurement between 5 and 10 years and one measurement between 10 and 18 years.

Between‐cohort differences in participant characteristics were tested using Student's *t*‐test or chi‐squared test.

Body mass index was log‐normally distributed in all cohorts. Therefore, we modelled log‐transformed BMI (lnBMI) in this study. LnBMI trajectories were modelled using multilevel models (two levels: measurement occasion and individual) in the statistical package MLwiN 10, using fractional polynomials to identify the best fitting curves. A model predicting ln(BMI) by age, age^2^ and age^3^ as both fixed and random effects, with age centered at 9 years, fitted the data within each of the cohorts well. The multilevel model allows for the change in scale and variance of BMI over time and allows measurement error to differ according to whether measurements are from questionnaires or from routine healthcare records or research examinations. We assumed that random variation was the same across all sources. We included an indicator of measurement source (questionnaires, routine healthcare records or research examinations) in the multilevel model as a fixed effect. The models use all available data for the study population under a missing at random assumption.

Differences in BMI trajectories between boys and girls, and between cohorts, were estimated by fitting interaction terms between gender/cohort and the constant term and each of the polynomial terms.

To assess model fit, we calculated mean BMI in the observed data at four different ages: 2 years (mean of all measurements between 2 and 3 years), 5 years (measurements between 4 and 6 years), 10 years (measurements between 9 and 11 years) and 15 years (measurements between 14 and 16 years).

To quantify the cohort differences in BMI, the mean predicted BMI in NFBC1986 and the mean per cent difference from this for the other cohorts were calculated. *Z*‐tests were used to assess the statistical evidence for the differences in BMI comparing the other cohorts to NFBC1986. To explore BMI differences at different stages of the obesity epidemic, we compared the two NFBC's, and to explore BMI differences between different populations, we compared NFBC1986, ABC and ALSPAC (all born within a span of 7 years).

As a sensitivity analysis, the model was fitted using all available data in order to check, whether the restriction to children with at least one measurement in each age interval resulted in changes compared with the full data set.

In order to explore potential explanations for cohort‐differences in BMI trajectories, we repeated our analyses controlling for a number of variables. Maternal height (cm), maternal BMI before pregnancy (kg/m^2^) and maternal smoking in the first trimester of pregnancy (yes/no) were included in the model, along with maternal age (<25, ≥25–<35 and ≥35 years of age), being firstborn (yes/no), maternal education (education longer than vocational level (courses designed to equip students with skills required for specific occupations) yes/no) and single parenthood (yes/no).

In order to investigate whether the differences in mean BMI, found between the cohorts, were due to a general increase in BMI in the whole population or a change in the tail of the distribution, centiles are reported and histograms of observed BMI at the age of 2, 5, 10 and 15 years were drawn. In the histograms, each child contributes a maximum of one measurement in each age group.

To explore height differences between the cohorts, centiles of height and weight at the age of 15 years were calculated.

## Results

Descriptive statistics are presented in Table 1. Birth weight was highest in the NFBC1986. Maternal BMI was highest in NFBC1966. The percentage of mothers smoking in pregnancy varied substantially from 13% in NFBC1966 to 29% in ABC. Also maternal education level varied from 15% having an education higher than vocational level in NFBC1966 to 78% in ALSPAC.

**Table 1:** Participant characteristics in NFBC1966, NFBC1986, ABC and ALSPAC

	NFBC1966 (N = 3321)			NFBC1986 (N = 4764)		ABC (N = 1920)			ALSPAC (N = 6517)		
	Mean (SD)	N (%)	*P*	Mean (SD)	N (%)	Mean (SD)	N (%)	*P*	Mean (SD)	N (%)	*P*
Birth weight [g]	3533 (480)	3321 (100)	<0.01	3627 (470)	4764 (98)	3551 (518)	1695 (88)	<0.01	3491 (467)	6461 (99)	<0.01
Maternal height [cm]	160 (5.3)	3169 (95)	<0.01	163 (5.4)	4723 (99)	168 (5.9)	1756 (91)	<0.01	164 (6.6)	6202 (95)	<0.01
Maternal BMI [kg/m2]	23.1 (3.2)	3056 (92)	<0.01	22.3 (3.4)	4660 (98)	21.7 (3.4)	1730 (90)	<0.01	22.9 (3.7)	5903 (91)	<0.01
Maternal age [years]	27.9 (6.5)	3315 (100)	<0.01	28.5 (5.4)	4764 (100)	30.1 (4.4))	1754 (91)	<0.01	29.1 (4.6)	6516 (100)	<0.01
	n (%)	N (%)	*P*	n (%)	N (%)	n (%)	N (%)	*P*	n (%)	N (%)	*P*
Offspring boy	1628 (49)	3321 (100)	0,83	2347 (49)	4764 (100)	986 (51)	1920 (100)	0,12	3259 (50)	6517 (100)	0,44
Maternal smoking	421 (13)	3254 (98)	<0.01	829 (18)	4664 (98)	435 (29)	1509 (79)	<0.01	1630 (26)	6386 (98)	<0.01
Maternal cohabiting	3215 (97)	3317 (100)	0,01	4559 (96)	4755 (100)	1479 (96)	1546 (81)	0,72	5982 (95)	6321 (97)	<0.01
Maternal education	487 (14.9)	3273 (99)	<0.01	1278 (30)	4199 (88)	1224 (66)	1868 (97)	<0.01	4890 (77.7)	6290 (97)	<0.01
Offspring first born	1138 (34)	3315 (100)	0,24	1570 (33)	4748 (100)	787 (50)	1570 (82)	<0.01	2933 (47)	6293 (97)	<0.01

N (%) refers to number of individuals in the study population with information on the variable and the percentage this constitutes of the given cohort.

ABC, the Aarhus Birth Cohort [{measured a median of 10 times (5–95% range: 6–13}]; ALSPAC, the Avon Longitudinal Study of Parents and Children [{measured a median of 9 times (5–95% range: 4–15}]; BMI, body mass index; NFBC1966, the Northern Finland Birth Cohort born 1966 [{measured a median of 14 times (5–95% range: 7–23}]; NFBC1986, the Northern Finland Birth Cohort born 1986 [{measured a median of 12 times (5–95% range: 8–17}]; SD, standard deviation.

At the age of 2 years, BMI in all the cohorts was approximately normally distributed. With increasing age, the distribution becomes more right skewed for all the cohorts. The BMI distribution also revealed that the cohorts, in spite of differences in the right tail of the distribution, contain a large proportion of individuals with very similar BMI across cohorts; density peaks and median BMI were seen at a similar BMI for all the cohorts (Supporting Information Figures S1–S4 and Table S1).

Our multilevel fractional polynomial model fits the data well, as the observed measurements of BMI across all cohorts, and all age groups are consistent with those predicted by the model (Supporting Information Table S2).

**Figure 1:**
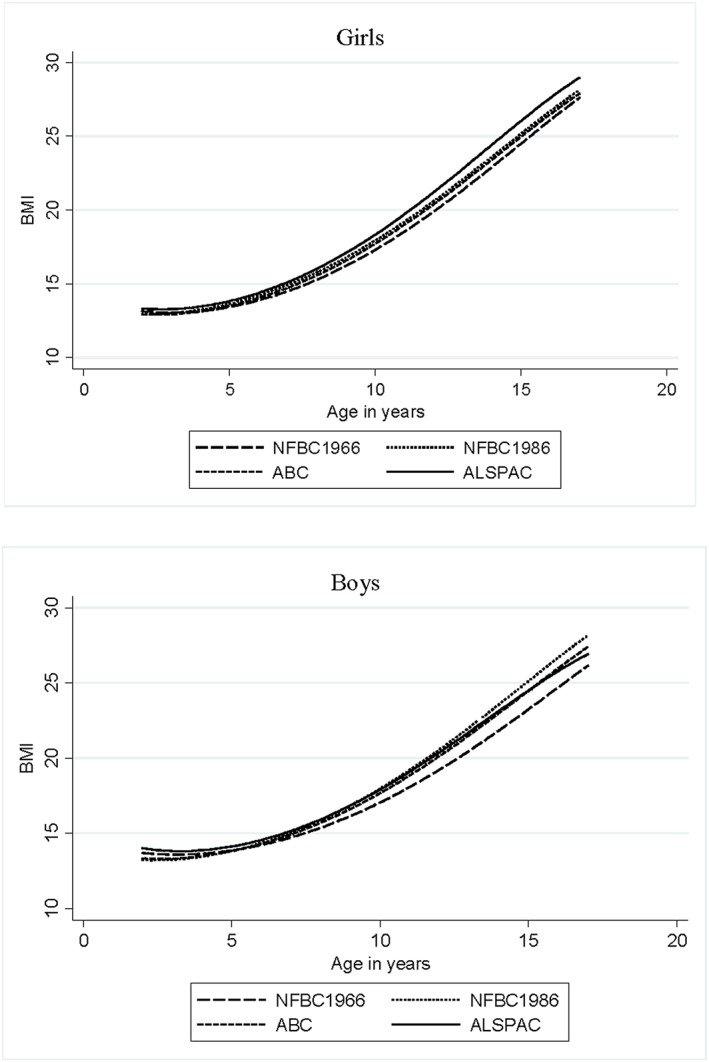
Average fractional polynomial curve of body mass index (BMI) trajectories for girls and boys from 2 to 17 years of age by cohort.

Mean predicted BMI trajectories in all four cohorts are depicted in Fig. 1. Table 2 shows the mean predicted BMI at the age of 2, 5, 10 and 15 years in NFBC1986 and the mean differences between NFBC1986 and the other cohorts. The largest difference between the cohorts was less than 6%. NFBC1966 had a higher mean BMI than NFBC1986 only at the age of 2 years (0.7–1.5%). After this age, the mean BMI in NFBC1986 exceeded that in NFBC1966 by 1.4–5.2%. BMI was 0.2–1.5% lower in ABC than in NFBC1986 throughout childhood in both genders. Girls in ALSPAC had a higher mean BMI (2.2–3.5% across childhood) than girls in NFBC1986. The ALSPAC boys had a 3.5% higher BMI than NFBC1986 boys at the age of 2 years but a similar BMI at ages 10 and 15 years.

**Table 2:** Mean predicted BMI in NFBC1986 and the mean difference from this for NFBC1966, ABC and ALSPAC (unadjusted for covariates)

	Mean predicted BMI (SD) in the NFBC1986	Mean % difference (CI), *p* between NFBC1966 and NFBC1986	Mean % difference (CI), *p* between ABC and NFBC1986	Mean % difference (CI), *p* between ALSPAC and NFBC1986
Girls				
2 years	16.4 (1.1)	1.5 (1.0 to 2.0), <0.001	−0.2 (−0.8 to 0.5), 0.572	2.9 (2.4 to 3.4), <0.001
5 years	15.7 (1.4)	−1.6 (−2.1 to −1.1), <0.001	−1.1 (−1.7 to −0.5), 0.001	1.3 (0.8 to 1.7), <0.001
10 years	17.5 (2.5)	−3.5 (−4.3 to −2.7), <0.001	−1.2 (−2.2 to −0.2), 0.023	2.2 (1.5 to 2.9), <0.001
15 years	20.8 (3.0)	−2.7 (−3.5 to−1.8), <0.001	−0.8 (−1.9 to 0.3), 0.156	3.5 (2.7 to 4.2), <0.001
Boys				
2 years	16.7 (1.1)	0.7 (0.2 to 1.3), 0.05	−0.4 (−1.0 to 0.6), 0.248	3.5 (3.0 to 4.0), <0.001
5 years	15.8 (1.3)	−1.4 (−1.9 to −0.9), <0.001	−0.8 (−1.4 to −0.1), 0.015	0.8 (0.3 to 1.2), 0.001
10 years	17.5 (2.5)	−4.7 (−5.5 to −3.9), <0.001	−1.5 (−2.4 to −0.5), 0.004	0.0 (−0.7 to 0.7), 0.992
15 years	20.9 (3.3)	−5.2 (−6.0 to −4.3), <0.001	−1.4 (−2.6 to −0.5), 0.005	−0.5 (−1.3 to 0.2), 0.170

*P* values are from *Z*‐tests comparing each of the other cohorts to NFBC1986.

ABC, the Aarhus Birth Cohort; ALSPAC, the Avon Longitudinal Study of Parents and Children; BMI, body mass index; CI, confidence interval; NFBC1966, the Northern Finland Birth Cohort born 1966; NFBC1986, the Northern Finland Birth Cohort born 1986; SD, standard deviation.

Including the full data did not change the results (Supporting Information Table S3).

Adjustment for maternal height, BMI and smoking revealed that NFBC1986 exceeded ALSPAC in mean BMI among males after the age of 5 years (Supporting Information Table S4). This was mainly driven by the adjustment for maternal BMI. Further adjustment for maternal age, being firstborn, maternal education and single parenthood did not essentially change the results.

Examining height separately at the age of 15 years revealed that ABC was taller than the other cohorts (Supporting Information Table S6).

## Discussion

### Main findings

In this study, we compared BMI trajectories in participants of four cohorts; two from Northern Finland, born in 1966 and 1986, one from Aarhus, Denmark, born in 1990–1992 and one from the Bristol area, United Kingdom, born in 1991–1993.

Overall, the differences in mean BMI between the cohorts were small. These small differences, however, emerged at a very early age and persisted in most cases across childhood.

Girls in ALSPAC had a higher BMI than all other cohorts throughout childhood, e.g. compared with NFBC1986, BMI was 1.3–3.5% higher. Girls in NFBC1966 had lower BMI than girls in the NFBC1986 from the age of 5 years. For boys, the difference emerging over time (comparing the two NFBC's) exceeded the differences across populations (comparing NFBC1986, ABC and ALSPAC).

### Comparison with other studies

Gender differences have also been detected in other studies 11. The increase in prevalence of overweight over time, seen here between NFBC1966 and NFBC1986, has been frequently reported using cross‐sectional data 2, 4, 12. Danish data have shown that the obesity prevalence among children and young adults has increased markedly with increasing year of birth but also that this increase in prevalence has developed in phases of increase and stagnation, for example, a steep increase in obesity prevalence between children born in 1966 and children born in 1986 was seen 12. This is in line with our finding on the Finnish data; a substantial increase in mean BMI from the age of 5 years between children born in 1966 and 1986. A study of worldwide trends in childhood overweight and obesity revealed that England and Finland also experienced an increase in overweight over the two last decades of the previous century 2. Newer data suggest a later levelling off of the obesity epidemic 13, at least in some countries 14. We cannot rule out that a levelling off of the obesity prevalence could have an impact on the younger cohorts, decreasing the overweight prevalence.

At age 2 years, the mean BMI in NFBC1966 was slightly higher than in NFBC1986, but after about the age of 3 years, mean BMI in NFBC1986 exceeds that in NFBC1966. On the individual level, we have previously found that children with a BMI among the highest 10% of a population at the age of 2 years are at increased risk of later overweight both in NFBC1966 and NFBC1986 18, 19, 20. Hence, it seems that children from a cohort of higher BMI at the age of 2 years are at lower risk of later overweight, but children in the high end of BMI, regardless of cohort, are at higher risk.

Whether the early differences in mean BMI between the cohorts are linked to later BMI, needs to be investigated further.

The remarkably stable growth patterns across different populations have been found in former studies 15, 16, but few have been able to analyze longitudinal data 17. Common features in the development of the society in these countries may have contributed to the similarities found e.g. they are all high‐income countries, with relatively stable political and economic systems. However, differences in local environment factors, policies, dietary behaviours, economic development, infant feeding 21, 22 or physical activity 23, 24 may at least partly explain the differences found.

The BMI distribution in the cohorts at the different ages was increasingly skewed to the right with increasing age, but the BMI was similar at the highest density in all four cohorts. Increasing overweight prevalence over time, without corresponding changes in the central part of the BMI distribution, has been shown before 25, 26.

We found the other cohorts to have slightly lower birth weight than NFBC1986, but the differences cannot be explained by the measured variables.

### Strengths and limitations

A major strength of this study was the prospective data collection in four large population‐based cohorts, with extensive measurements of height and weight from childhood to adolescence. One limitation was the variation in the ages and mode of collection of BMI measurements, but we believe the differences were adequately handled by the use of the multilevel model. A large proportion of the study population was excluded when requiring three measurements; especially the study population of the ABC was reduced by 70%. The major explanation for missing measurements in the ABC was the national execution of a political decision to change municipal organization just prior to data collection and hence inaccessible archives. We have no reason to believe that this caused a selective reduction and thereby was a source of selection bias, and this is borne out by our sensitivity analyses. We know from other analyses of NFBC1966 and ALSPAC that individuals with only basic education and individuals with unemployment history are slightly under‐represented among attendees 27, 28 and that individuals with many BMI measurements in the NFBC1966 have slightly lower adult BMI than individuals with fewer measurements 18. If this has an impact on the results, we will most likely have underestimated the mean BMI, as the study participants are expected to be slightly leaner than the source population, but we expect that this applies equally to all four cohorts. Moreover, we saw substantial differences in maternal education between the cohorts and adjusted for this, but because of differences in the educational systems between Denmark, Finland and United Kingdom the information obtained on maternal education level may not have been sufficiently comparable to fully adjust for the effect of maternal education.

## Conclusion

Population‐level differences between cohorts were small, tended to emerge at a very early age, persisted across childhood and demonstrated an increase in the right‐hand tail of the BMI distribution with increasing age.

## Supporting information


**Figure S1.** Histogram of actual BMI at the age of 2 years
**Figure S2.** Histogram of actual BMI at the age of 5 years
**Figure S3.** Histogram of actual BMI at the age of 10 years
**Figure S4.** Histogram of actual BMI at the age of 15 years
**Table S1.** BMI at 5^th^, 50^th^ and 95^th^ percentile at the age of 2, 5, 10 and 15 years by gender and cohort
**Table S2.** Means of observed BMI and BMI predicted from the multilevel model with their standard deviation (SD)
**Table S3.** Mean predicted BMI in NFBC1986 and the mean difference from this for NFBC1966, ABC and ALSPAC on all available data (not restricted to children with at least 3 measurements)
**Table S4.** Mean predicted BMI in NFBC1986 and the mean difference from this for NFBC1966, ABC and ALSPAC (adjusted for maternal height, maternal BMI and smoking during pregnancy)
**Table S5.** Mean predicted BMI in the NFBC1986 and the mean difference from this for NFBC1966, ABC and ALSPAC (adjusted for maternal height, maternal BMI, smoking during pregnancy, being firstborn, single parenthood, maternal education and maternal age)
**Table S6.** Height and weight at 5^th^, 50^th^ and 95^th^centiles at the age of 15 years

Supporting info item

## References

[1] Ng M , Fleming T , Robinson M , *et al.* Global, regional, and national prevalence of overweight and obesity in children and adults during 1980‐2013: a systematic analysis for the Global Burden of Disease Study 2013. Lancet 2014; 28: 766–781. 10.1016/S0140-6736(14)60460-8 PMC4624264 24880830

[2] Wang Y , Lobstein T . Worldwide trends in childhood overweight and obesity. Int J Pediatr Obes 2006; 1: 11–25. 17902211 10.1080/17477160600586747

[3] Lobstein T , Baur L , Uauy R , IASO International Obesity TaskForce. Obesity in children and young people: a crisis in public health. Obes Rev 2004; 5(Suppl 1):4–104 15096099 10.1111/j.1467-789X.2004.00133.x

[4] de Onis M , Blossner M , Borghi E . Global prevalence and trends of overweight and obesity among preschool children. Am J Clin Nutr 2010; 92: 1257–1264. 20861173 10.3945/ajcn.2010.29786

[5] Janssen I , Katzmarzyk PT , Boyce WF , *et al.* Comparison of overweight and obesity prevalence in school‐aged youth from 34 countries and their relationships with physical activity and dietary patterns. Obes Rev 2005; 6: 123–132. 15836463 10.1111/j.1467-789X.2005.00176.x

[6] Rantakallio P . Groups at risk in low birth weight infants and perinatal mortality. Acta Paediatr Scand 1969; 193(Suppl 193): 1+. 4911003

[7] Jarvelin MR , Sovio U , King V , *et al.* Early life factors and blood pressure at age 31 years in the 1966 northern Finland birth cohort. Hypertension 2004; 44: 838–846. 15520301 10.1161/01.HYP.0000148304.33869.ee

[8] Hedegaard M , Henriksen TB , Sabroe S , Secher NJ . Psychological distress in pregnancy and preterm delivery. BMJ 1993; 307: 234–239. 8369684 10.1136/bmj.307.6898.234 PMC1678180

[9] Boyd A , Golding J , Macleod J , *et al.* Cohort Profile: the ‘children of the 90s’–the index offspring of the Avon Longitudinal Study of Parents and Children. Int J Epidemiol 2013; 42: 111–127. 22507743 10.1093/ije/dys064 PMC3600618

[10] Rasbash J , Charlton C , Browne WJ , Healy M , Cameron B . *MLwiN Version 2.1*. Centre for Multilevel Modelling. University of Bristol, 2009 www.bristol.ac.uk/cmm/software/mlwin/download/manuals.html.

[11] Lundeen EA , Norris SA , Adair LS , Richter LM , Stein AD . Sex differences in obesity incidence: 20‐year prospective cohort in South Africa. Pediatr Obes 2016; 11 (1): 75–80. 25988503 10.1111/ijpo.12039 PMC4832364

[12] Olsen LW , Baker JL , Holst C , Sorensen TI . Birth cohort effect on the obesity epidemic in Denmark. Epidemiology 2006; 17: 292–295. 16570023 10.1097/01.ede.0000208349.16893.e0

[13] Rokholm B , Baker JL , Sorensen TI . The levelling off of the obesity epidemic since the year 1999–a review of evidence and perspectives. Obes Rev 2010; 11: 835–846. 20973911 10.1111/j.1467-789X.2010.00810.x

[14] Wijnhoven TM , van Raaij JM , Spinelli A , *et al.* WHO European Childhood Obesity Surveillance Initiative: body mass index and level of overweight among 6‐9‐year‐old children from school year 2007/2008 to school year 2009/2010. BMC Public Health 2014; 7: 806‐2458–14‐806. 10.1186/1471-2458-14-806 PMC4289284 25099430

[15] Cole TJ , Bellizzi MC , Flegal KM , Dietz WH . Establishing a standard definition for child overweight and obesity worldwide: international survey. BMJ 2000; 320: 1240–1243. 10797032 10.1136/bmj.320.7244.1240 PMC27365

[16] WHO Multicentre Growth Reference Study Group . Assessment of differences in linear growth among populations in the WHO Multicentre Growth Reference Study. Acta Paediatr Suppl 2006; 450: 56–65. 16817679 10.1111/j.1651-2227.2006.tb02376.x

[17] Howe LD , Tilling K , Matijasevich A , *et al.* Linear spline multilevel models for summarising childhood growth trajectories: a guide to their application using examples from five birth cohorts. Stat Methods Med Res 2013; 9. 10.1177/0962280213503925 PMC4074455 24108269

[18] Graversen L , Sorensen TI , Petersen L , *et al.* Preschool weight and body mass index in relation to central obesity and metabolic syndrome in adulthood. PLoS One 2014; 9: e89986. 24595022 10.1371/journal.pone.0089986 PMC3940896

[19] Graversen L , Sorensen TI , Petersen L , *et al.* Stability of the associations between early life risk indicators and adolescent overweight over the evolving obesity epidemic. PLoS One 2014; 9: e95314. 24748033 10.1371/journal.pone.0095314 PMC3991687

[20] Sovio U , Kaakinen M , Tzoulaki I , Das S , Ruokonen A , Pouta A , *et al.* How do changes in body mass index in infancy and childhood associate with cardiometabolic profile in adulthood? Findings from the Northern Finland Birth Cohort 1966 Study. Int J Obes (Lond) 2014; 38(1): 53–59. 24080793 10.1038/ijo.2013.165

[21] Harder T , Bergmann R , Kallischnigg G , Plagemann A . Duration of breastfeeding and risk of overweight: a meta‐analysis. Am J Epidemiol 2005; 162: 397–403. 16076830 10.1093/aje/kwi222

[22] Monasta L , Batty GD , Cattaneo A , *et al.* Early‐life determinants of overweight and obesity: a review of systematic reviews. Obes Rev 2010; 11(10); 695–708. 20331509 10.1111/j.1467-789X.2010.00735.x

[23] Li R , O'Connor L , Buckley D , Specker B . Relation of activity levels to body fat in infants 6 to 12 months of age. J Pediatr 1995; 126: 353–357. 7869191 10.1016/s0022-3476(95)70447-7

[24] Sijtsma A , Sauer PJ , Stolk RP , Corpeleijn E . Is directly measured physical activity related to adiposity in preschool children? Int J Pediatr Obes 2011; 6: 389–400. 21834604 10.3109/17477166.2011.606323

[25] Thomsen BL , Ekstrom CT , Sorensen TI . Development of the obesity epidemic in Denmark: cohort, time and age effects among boys born 1930‐1975. Int J Obes Relat Metab Disord 1999; 23: 693–701. 10454102 10.1038/sj.ijo.0800907

[26] Christensen U , Sonne‐Holm S , Sorensen TI . Constant median body mass index of Danish young men, 1943–1977. Hum Biol 1981; 53: 403–410. 7309025

[27] Sovio U , King V , Miettunen J , *et al.* Cloninger's temperament dimensions, socio‐economic and lifestyle factors and metabolic syndrome markers at age 31 years in the Northern Finland Birth Cohort 1966. J Health Psychol 2007; 12: 371–382. 17284500 10.1177/1359105307074301

[28] Howe LD , Tilling K , Galobardes B , Lawlor DA . Loss to follow‐up in cohort studies: bias in estimates of socioeconomic inequalities. Epidemiology 2013; 24: 1–9. 23211345 10.1097/EDE.0b013e31827623b1 PMC5102324

