# Supplementary material for: Body mass index trajectories from 2 to 18 years – exploring differences between European cohorts
Source: Pediatr Obes. 2016 Feb 26;12(2):102–9. doi: 10.1111/ijpo.12115 (PMC5347959; doi:10.1111/ijpo.12115)
Supplement: Supplementary file 1 — Supporting info item [file IJPO-12-102-s001.zip › Supplementary table 1 30_09_2015.docx]

**Supplementary table 1:** BMI at 5^th^, 50^th^ and 95^th^ percentile at the age of 2, 5, 10 and 15 years by gender and cohort.

| Girls |  | NFBC1966 | |  | NFBC1986 | |  | ABC | |  | ALSPAC | |
| --- | --- | --- | --- | --- | --- | --- | --- | --- | --- | --- | --- | --- |
| Age | Percentile | BMI | 95% CI |  | BMI | 95% CI |  | BMI | 95% CI |  | BMI | 95% CI |
| 2 years | 5 | 14.32 | 14.20-14.45 |  | 14.39 | 14.30-14.49 |  | 14.20 | 14.03-14.41 |  | 14.24 | 14.08-14.41 |
|  | 50 | 16.41 | 16.31-16.48 |  | 16.32 | 16.25-16.38 |  | 16.41 | 16.24-16.51 |  | 16.30 | 16.22-16.41 |
|  | 95 | 18.76 | 18.60-18.94 |  | 18.69 | 18.51-18.87 |  | 18.53 | 18.35-18.77 |  | 18.75 | 18.62-18.97 |
|  |  |  |  |  |  |  |  |  |  |  |  |  |
| 5 Years | 5 | 13.45 | 13.30-15.57 |  | 13.68 | 13.59-13.79 |  | 13.54 | 13.39-13.65 |  | 13.02 | 12.77-13.19 |
|  | 50 | 15.29 | 15.15-15.42 |  | 15.52 | 15.45-15.58 |  | 15.23 | 15.11-15.37 |  | 15.61 | 15.51-15.72 |
|  | 95 | 18.13 | 17.80-18.74 |  | 18.27 | 18.09-18.40 |  | 17.54 | 17.24-18.09 |  | 18.57 | 18.47-18.99 |
|  |  |  |  |  |  |  |  |  |  |  |  |  |
| 10 years | 5 | 14.10 | 13.94-14.31 |  | 14.40 | 14.14-14.53 |  | 14.28 | 13.86-14.53 |  | 14.41 | 14.28-14.49 |
|  | 50 | 16.62 | 16.47-16.78 |  | 17.19 | 17.04-17.36 |  | 17.09 | 16.75-17.39 |  | 17.63 | 17.49-17.75 |
|  | 95 | 22.14 | 21.69-23.16 |  | 23.74 | 23.09-24.30 |  | 22.64 | 21.74-24.12 |  | 24.45 | 23.93-25.06 |
|  |  |  |  |  |  |  |  |  |  |  |  |  |
| 15 years | 5 | 16.89 | 16.57-17.16 |  | 17.13 | 16.95-17.34 |  | 16.88 | 16.67-17.14 |  | 17.04 | 16.92-17.27 |
|  | 50 | 19.95 | 19.95-20.19 |  | 20.32 | 20.12-20.51 |  | 20.31 | 20.11-20.62 |  | 20.96 | 20.81-21.11 |
|  | 95 | 26.39 | 25.37-27.33 |  | 27.12 | 26.31-27.92 |  | 25.10 | 24.54-25.71 |  | 28.20 | 27.55-28.82 |
|  |  |  |  |  |  |  |  |  |  |  |  |  |
| Boys |  |  | |  |  | |  |  | |  |  | |
| 2 years | 5 | 14.49 | 14.27-14.67 |  | 14.67 | 14.57-14.80 |  | 14.52 | 14.29-14.77 |  | 14.60 | 14.45-14.81 |
|  | 50 | 16.54 | 16.42-16.62 |  | 16.48 | 16.41-16.55 |  | 16.51 | 16.41-16.61 |  | 16.65 | 16.55-16.72 |
|  | 95 | 18.95 | 18.83-19.24 |  | 18.77 | 18.66-18.94 |  | 18.94 | 18.72-19.27 |  | 18.98 | 18.72-19.31 |
|  |  |  |  |  |  |  |  |  |  |  |  |  |
| 5 Years | 5 | 13.72 | 13.55-13.88 |  | 13.88 | 13.80-13.97 |  | 13.51 | 13.36-13.66 |  | 13.41 | 13.19-13.44 |
|  | 50 | 15.39 | 15.29-15.45 |  | 15.58 | 15.53-15.66 |  | 15.39 | 15.26-15.53 |  | 15.66 | 15.60-15.72 |
|  | 95 | 17.66 | 17.46-17.88 |  | 18.05 | 18.05-18.57 |  | 17.86 | 17.58-18.27 |  | 18.64 | 18.44-18.90 |
|  |  |  |  |  |  |  |  |  |  |  |  |  |
| 10 years | 5 | 14.47 | 14.31-14.60 |  | 14.61 | 14.49-14.73 |  | 14.43 | 14.27-14.58 |  | 14.59 | 14.46-14.69 |
|  | 50 | 16.57 | 16.44-16.69 |  | 17.30 | 17.17-17.37 |  | 16.99 | 16.71-17.22 |  | 17.23 | 17.09-17.33 |
|  | 95 | 21.04 | 20.37-21.73 |  | 23.44 | 22.87-24.23 |  | 22.80 | 21.85-23.69 |  | 23.77 | 23.43-24.24 |
|  |  |  |  |  |  |  |  |  |  |  |  |  |
| 15 years | 5 | 16.74 | 16.743-16.88 |  | 16.75 | 16.48-16.94 |  | 17.19 | 16.73-17.33 |  | 17.02 | 16.82-17.18 |
|  | 50 | 19.49 | 19.30-19.63 |  | 20.27 | 20.06-20.43 |  | 20.38 | 20.09-20.60 |  | 20.24 | 20.11-20.44 |
|  | 95 | 24.42 | 23.82-25.35 |  | 28.51 | 27.17-29.69 |  | 26.82 | 26.20-27.65 |  | 27.60 | 26.89-28.06 |

Footnote:

NFBC1966: The Northern Finland Birth Cohort born 1966

NFBC1986: The Northern Finland Birth Cohort born 1986

ABC: The Aarhus Birth Cohort

ALSPAC: The Avon Longitudinal Study of Parents and Children
