# Supplementary material for: Body mass index trajectories from 2 to 18 years – exploring differences between European cohorts
Source: Pediatr Obes. 2016 Feb 26;12(2):102–9. doi: 10.1111/ijpo.12115 (PMC5347959; doi:10.1111/ijpo.12115)
Supplement: Supplementary file 1 — Supporting info item [file IJPO-12-102-s001.zip › Supplementary table 2.docx]

# Supplementary table 2: Means of observed BMI and BMI predicted from the multilevel model with their standard deviation (SD).

|  |  | NFBC1966 | | |  | NFBC1986 | | |  | ABC | | |  | ALSPAC | | |
| --- | --- | --- | --- | --- | --- | --- | --- | --- | --- | --- | --- | --- | --- | --- | --- | --- |
| Girls |  | N= 1693 | | |  | N=2417 | | |  | N=934 | | |  | N=3258 | | |
|  |  |  | Mean observed BMI (SD) | Mean predicted BMI (SD) |  |  | Mean observed BMI (SD) | Mean predicted BMI (SD) |  |  | Mean observed BMI (SD) | Mean predicted BMI (SD) |  |  | Mean observed BMI (SD) | Mean predicted BMI (SD) |
| Age |  | n |  |  |  | n |  |  |  | n |  |  |  | n |  |  |
|  |  |  |  |  |  |  |  |  |  |  |  |  |  |  |  |  |
| 2 |  | 2003 | 16.4(1.4) | 16.4(1.2) |  | 2452 | 16.3(1.3) | 16.3(1.1) |  | 835 | 16.4(1.4) | 16.3(1.1) |  | 1539 | 16.4(1.4) | 16.5(1.1) |
| 5 |  | 2996 | 15.5(1.5) | 15.5(1.3) |  | 4493 | 15.7(1.4) | 15.7(1.3) |  | 1464 | 15.5(1.4) | 15.5(1.2) |  | 3419 | 15.8(1.7) | 15.9(1.4) |
| 10 |  | 2494 | 17.0(2.6) | 17.0(2.5) |  | 3681 | 17.6(2.8) | 17.5(2.6) |  | 918 | 17.3(2.5) | 17.2(2.3) |  | 5898 | 18.0(3.0) | 18.0(2.9) |
| 15 |  | 2734 | 20.2(3.0) | 20.0(2.9) |  | 3356 | 20.6(3.2) | 20.6(3.1) |  | 1344 | 20.4(2.7) | 20.3(2.6) |  | 2490 | 21.4(3.5) | 21.4(3.3) |
|  |  |  |  |  |  |  |  |  |  |  |  |  |  |  |  |  |
| Boys |  | N= 1628 | | |  | N=2347 | | |  | N=986 | | |  | N=3259 | | |
|  |  |  | Mean observed BMI (SD) | Mean predicted BMI (SD) |  |  | Mean observed BMI (SD) | Mean predicted BMI (SD) |  |  | Mean observed BMI (SD) | Mean predicted BMI (SD) |  |  | Mean observed BMI (SD) | Mean predicted BMI (SD) |
| Age |  | n |  |  |  | n |  |  |  | n |  |  |  | n |  |  |
|  |  |  |  |  |  |  |  |  |  |  |  |  |  |  |  |  |
| 2 |  | 1948 | 16.6(1.4) | 16.6(1.1) |  | 2406 | 16.6(1.3) | 16.6(1.1) |  | 830 | 16.6(1.4) | 16.5(1.1) |  | 1615 | 16.7(1.3) | 16.8(1.1) |
| 5 |  | 2683 | 15.6(1.2) | 15.6(1.1) |  | 4326 | 15.9(1.5) | 15.9(1.3) |  | 1488 | 15.6(1.4) | 15.7(1.2) |  | 3608 | 15.9(1.6) | 15.9(1.3) |
| 10 |  | 2307 | 16.8(2.1) | 16.7(1.9) |  | 3377 | 17.7(2.8) | 17.6(2.7) |  | 995 | 17.5(2.6) | 17.4(2.5) |  | 5692 | 17.7(2.8) | 17.7(2.6) |
| 15 |  | 2724 | 19.5(2.5) | 19.5(2.4) |  | 3071 | 20.9(3.7) | 20.9(3.6) |  | 1422 | 20.6(3.1) | 20.6(2.9) |  | 2342 | 20.7(3.3) | 20.7(3.1) |
|  |  |  |  |  |  |  |  |  |  |  |  |  |  |  |  |  |

| Footnote:  N= studypopulation |  |  |  |  |  |  |  |  |  |  |  |  |  |
| --- | --- | --- | --- | --- | --- | --- | --- | --- | --- | --- | --- | --- | --- |
| n= number of observations the mean observed and predicted BMI are based on. A child can contribute with more than one measurement at the given ages. | | | | | | | | |  |  |  |  |  |
| NFBC1966: The Northern Finland Birth Cohort born 1966 | | | | | |  |  |  |  |  |  |  |  |
| NFBC1986: The Northern Finland Birth Cohort born 1986 | | | | | |  |  |  |  |  |  |  |  |
| ABC: The Aarhus Birth Cohort | |  |  |  |  |  |  |  |  |  |  |  |  |
| ALSPAC: The Avon Longitudinal Study of Parents and Children | | | | | |  |  |  |  |  |  |  |  |
| BMI: Body Mass Index  SD: Standard Deviation | | | | | |  |  |  |  |  |  |  |  |
