# Supplementary material for: Body mass index trajectories from 2 to 18 years – exploring differences between European cohorts
Source: Pediatr Obes. 2016 Feb 26;12(2):102–9. doi: 10.1111/ijpo.12115 (PMC5347959; doi:10.1111/ijpo.12115)
Supplement: Supplementary file 1 — Supporting info item [file IJPO-12-102-s001.zip › Supplementary table 3 30_9_2015.docx]

**Supplementary table 3:** Mean predicted BMI in NFBC1986 and the mean difference from this for NFBC1966, ABC and ALSPAC on all available data (not restricted to children with at least 3 measurements).

|  |  |  | |  |  |  |  | | |  |  | | |  |
| --- | --- | --- | --- | --- | --- | --- | --- | --- | --- | --- | --- | --- | --- | --- |
|  |  | Mean predicted BMI (SD) in the NFBC1986 |  | | Mean % difference (CI),p between NFBC1966 and NFBC1986 | | |  | Mean % difference (CI),p between ABC and NFBC1986 | | |  | Mean % difference (CI),p between ALSPAC and NFBC1986 | |
|  |  |  |  | |  | | |  |  | | |  |  | |
| Girls |  |  |  | |  |  |  |  |  |  |  |  |  |  |
| 2 years |  | 16.4(1.1) |  | | 1.5(1.0 to 2.1),<0.001 | | |  | -0.1(-0.7 to 0.5),0.775 | | |  | 2.8(2.2 to 3.2),<0.001 | |
| 5 years |  | 15.7(1.4) |  | | -1.7(-2.8 to -1.2),<0.001 | | |  | -0.8(-1.3 to -0.3),0.002 | | |  | 1.6(1.2 to 2.1),<0.001 | |
| 10 years |  | 17.5(2.5) |  | | -3.7(-4.5 to -3.0),<0.001 | | |  | -1.4(-2.1 to -0.6),0.001 | | |  | 2.7(2.1 to 3.4),<0.001 | |
| 15 years |  | 20.8(3.0) |  | | -2.8(-3.6 to -2.0),<0.001 | | |  | -0.7(-1.5 to -0.2),0.112 | | |  | 4.1(3.3 to 4.9),<0.001 | |
|  |  |  |  | |  | | |  |  | | |  |  | |
| Boys |  |  |  | |  | | |  |  | | |  |  | |
| 2 years |  | 16.7(1.1) |  | | 0.6(0.1 to 1.1),0.024 | | |  | -0.5(-1.0 to 0.1),0.130 | | |  | 3.3(2.9 to 3.8),<0.001 | |
| 5 years |  | 15.8(1.3) |  | | -1.4(-2.0 to -1.0),<0.001 | | |  | -0.5(-1.0 to 0.0),0.070 | | |  | 1.2(0.8 to 1.6),<0.001 | |
| 10 years |  | 17.5(2.5) |  | | -4.7(-5.4 to -3.9),<0.001 | | |  | -1.4(-2.2 to -0.6),0.001 | | |  | 0.6(-0.1 to 1.2),0.097 | |
| 15 years |  | 20.9(3.3) |  | | -5.3(-6.1 to -4.4),<0.001 | | |  | -1.2(-2.1 to -0.4),0.004 | | |  | 0.0(-0.8 to 0.7),0.945 | |
|  |  |  |  | |  | | |  |  | | |  |  | |
|  |  |  |  | |  |  |  | | |  |  | | |  |
|  |  |  | |  |  |  |  | | |  |  | | |  |

Footnote:

P values are from Z-tests comparing each of the other cohorts to NFBC1986.

NFBC1966: The Northern Finland Birth Cohort born 1966

NFBC1986: The Northern Finland Birth Cohort born 1986

ABC: The Aarhus Birth Cohort

ALSPAC: The Avon Longitudinal Study of Parents and Children

BMI: Body Mass Index

SD: Standard Deviation

CI: Confidence interval
