# Supplementary material for: Body mass index trajectories from 2 to 18 years – exploring differences between European cohorts
Source: Pediatr Obes. 2016 Feb 26;12(2):102–9. doi: 10.1111/ijpo.12115 (PMC5347959; doi:10.1111/ijpo.12115)
Supplement: Supplementary file 1 — Supporting info item [file IJPO-12-102-s001.zip › Supplementary table 4.docx]

# Supplementary table 4: Mean predicted BMI in NFBC1986 and the mean difference from this for NFBC1966, ABC and ALSPAC (adjusted for maternal height, maternal BMI and smoking during pregnancy).

|  |  | Mean predicted BMI (SD) in the NFBC1986 |  | Mean % difference (CI),p between NFBC1966 and NFBC1986 | |  | Mean % difference (CI),p between ABC and NFBC1986 | | | | | |  | Mean % difference (CI),p between ALSPAC and NFBC1986 | | | |
| --- | --- | --- | --- | --- | --- | --- | --- | --- | --- | --- | --- | --- | --- | --- | --- | --- | --- |
|  |  |  |  |  |  |  |  |  |  |  |  |  |  |  |  |  |  |
|  |  |  |  |  |  |  |  |  |  |  |  |  |  |  |  |  |  |
| Girls |  |  |  |  |  |  |  |  |  |  |  |  |  |  |  |  |  |
| 2 years |  | 16.5(1.1) |  | 1.5(0.9 to 2.0),<0.001 | |  | -0.2(-0.9 to 0.5),0.573) | | | | | |  | 2.7(2.2 to 3.2),<0.001 | | | |
| 5 years |  | 15.7(1.3) |  | -1.7(-2.2to -1.2),<0.001 | |  | -1.1(-1.8 to -0.5),0.001 | | | | | |  | 1.0(0.6 to 1.5),<0.001 | | | |
| 10 years |  | 17.5(2.4) |  | -4.0(-4.8 to -3.2),<0.001 | |  | -1.1(-2.2 to 0.0),0.043 | | | | | |  | 1.3(0.6 to 2.0),<0.001 | | | |
| 15 years |  | 20.8(2.8) |  | -3.3(-4.2 to -2.5),<0.001 | |  | -0.5(-1.6 to 0.7),0.431 | | | | | |  | 2.5(1.8 to 3.3),<0.001 | | | |
|  |  |  |  |  | |  |  | | | | | |  |  | | | |
| Boys |  |  |  |  | |  |  | | | | | |  |  | | | |
| 2 years |  | 16.7(1.1) |  | 0.6(0.0 to 1.1),0.049 | |  | -0.3(-1.0 to 0.4),0.377 | | | | | |  | 3.2(2.7 to 3.7) ,<0.001 | | | |
| 5 years |  | 15.8(1.3) |  | -1.6(-2.2 to -1.1),<0.001 | |  | -1.0(-1.7 to -0.3),0.004 | | | | | |  | 0.2(-0.3 to 0.7),0.391 | | | |
| 10 years |  | 17.6(2.4) |  | -5.2(-6.0 to -4.4),<0.001 | |  | -1.6(-2.6 to -0.6),0.003 | | | | | |  | -1.3(-2.0 to -0.6),<0.001 | | | |
| 15 years |  | 20.9(3.1) |  | -5.9(-6.8 to -5.1),<0.001 | |  | -1.2(-2.4 to -0.1),0.038 | | | | | |  | -2.1(-2.8 to -1.3),<0.001 | | | |
|  |  |  |  |  |  |  | | | | |  |  | | | | |  |
|  |  |  |  |  |  |  | | |  | |  | | | | |  |  |
|  |  |  |  |  |  |  | |  | |  | | | | |  |  |  |

Footnote:

The values represent the predicted BMI for offspring of non-smoking mothers with a BMI of 22 and a height of 163 cm.

P values are from Z-tests comparing each of the other cohorts to NFBC1986.

NFBC1966: The Northern Finland Birth Cohort born 1966

NFBC1986: The Northern Finland Birth Cohort born 1986

ABC: The Aarhus Birth Cohort

ALSPAC: The Avon Longitudinal Study of Parents and Children

BMI: Body Mass Index

SD: Standard Deviation

CI: Confidence Interval
