# Supplementary material for: Body mass index trajectories from 2 to 18 years – exploring differences between European cohorts
Source: Pediatr Obes. 2016 Feb 26;12(2):102–9. doi: 10.1111/ijpo.12115 (PMC5347959; doi:10.1111/ijpo.12115)
Supplement: Supplementary file 1 — Supporting info item [file IJPO-12-102-s001.zip › Supplementary table 5.docx]

**Supplementary table 5:** Mean predicted BMI in the NFBC1986 and the mean difference from this for NFBC1966, ABC and ALSPAC (adjusted for maternal height, maternal BMI, smoking during pregnancy, being firstborn, single parenthood, maternal education and maternal age).

|  |  | Mean predicted BMI (SD) in the NFBC1986 |  | Mean % difference (CI),p between NFBC1966 and NFBC1986 |  | Mean % difference (CI),p between ABC and NFBC1986 | | |  | Mean % difference (CI),p between ALSPAC and NFBC1986 | |
| --- | --- | --- | --- | --- | --- | --- | --- | --- | --- | --- | --- |
|  |  |  |  |  |  |  |  |  |  |  |  |
|  |  |  |  |  |  |  |  |  |  |  |  |
| Girls |  |  |  |  |  |  |  |  |  |  |  |
| 2 years |  | 16.5(1.1) |  | 1.5(0.9 to 2.1), <0.001 |  | -0.2(-1.0 to 0.5), 0.557 | | |  | 2.6(2.0 to 3.2), <0.001 | |
| 5 years |  | 15.8(1.3) |  | -1.7(-2.3 to -1.2), <0.001 |  | -1.3(-2.0 to -0.6), <0.001 | | |  | 0.8(0.3 to 1.3), 0.003 | |
| 10 years |  | 17.8(2.4) |  | -4.4(-5.0 to -3.4), <0.001 |  | -1.2(-2.3 to -0.1), 0.039 | | |  | 1.2(0.5 to 2.0), 0.002 | |
| 15 years |  | 21.2(2.9) |  | -3.7(-4.6 to -2.9), <0.001 |  | -0.3(-1.5 to 0.9), 0.579 | | |  | 2.7(1.8 to 3.6), <0.001 | |
|  |  |  |  |  |  |  | | |  |  | |
| Boys |  |  |  |  |  |  | | |  |  | |
| 2 years |  | 16.7(1.1) |  | 0.5(0.0 to 1.1), 0.059 |  | -0.4(-1.1 to 0.3), 0.287 | | |  | 3.0(2.4 to 3.6), <0.001 | |
| 5 years |  | 15.9(1.3) |  | -1.6(-2.2 to -1.1) ,<0.001 |  | -1.2(-1.9 to -0.4), 0.002 | | |  | -0.1(-0.6 to 0.4), 0.629 | |
| 10 years |  | 17.9(2.4) |  | -5.4(-6.2 to -4.6) , <0.001 |  | -1.6(-2.7 to -0.5), 0.006 | | |  | -1.4(-2.2 to -0.6), <0.001 | |
| 15 years |  | 21.3(3.2) |  | -6.3(-7.2 to-5.4), <0.001 |  | -1.0(-2.1 to 0.2), 0.119 | | |  | -1.9(-2.7 to -1.0), <0.001 | |
|  |  |  |  |  |  |  | | |  |  | |
|  |  |  |  |  |  |  |  |  | | |  |
|  |  |  |  |  |  |  |  |  | | |  |

Footnote:

The values represent the predicted BMI for firstborn offspring of non-smoking cohabiting mothers below 25 years of age with a BMI of 22, a height of 163 cm and with short education.

P values are from Z-tests comparing each of the other cohorts to NFBC1986.

NFBC1966: The Northern Finland Birth Cohort born 1966

NFBC1986: The Northern Finland Birth Cohort born 1986

ABC: The Aarhus Birth Cohort

ALSPAC: The Avon Longitudinal Study of Parents and Children

BMI: Body Mass Index

SD: Standard Deviation

CI: Confidence Interval
