# Supplementary material for: Body mass index trajectories from 2 to 18 years – exploring differences between European cohorts
Source: Pediatr Obes. 2016 Feb 26;12(2):102–9. doi: 10.1111/ijpo.12115 (PMC5347959; doi:10.1111/ijpo.12115)
Supplement: Supplementary file 1 — Supporting info item [file IJPO-12-102-s001.zip › Supplementary table 6 28_9-2015.docx]

**Supplementary table 6:** Height and weight at 5^th^, 50^th^ and 95^th^centiles at the age of 15 years.

Footnote:

NFBC1966: The Northern Finland Birth Cohort born 1966

NFBC1986: The Northern Finland Birth Cohort born 1986

ABC: The Aarhus Birth Cohort

ALSPAC: The Avon Longitudinal Study of Parents and Children
