# Supplementary figures and images for: Body mass index trajectories from 2 to 18 years – exploring differences between European cohorts
Source: Pediatr Obes. 2016 Feb 26;12(2):102–9. doi: 10.1111/ijpo.12115 (PMC5347959; doi:10.1111/ijpo.12115)

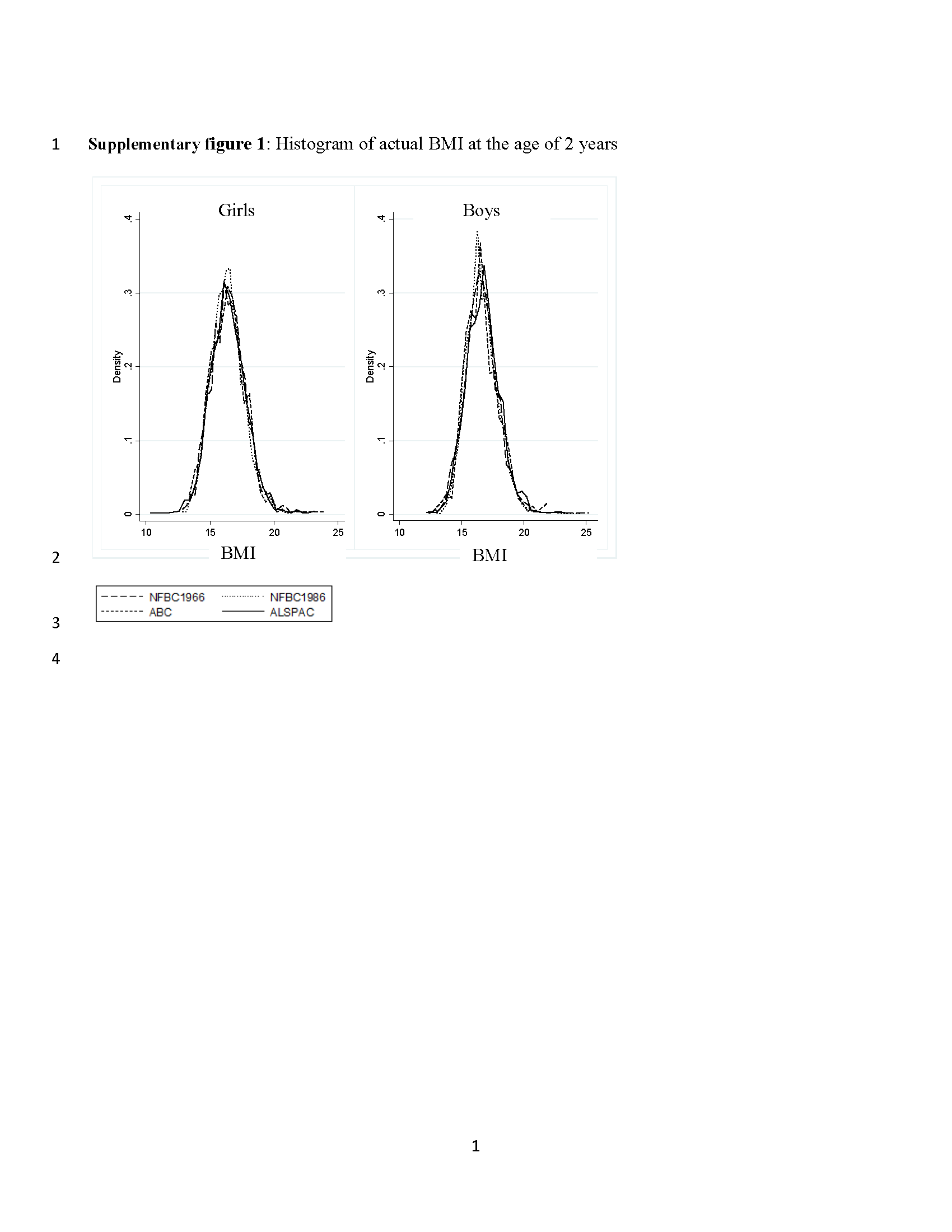

Supplement: Supplementary file 1 — Supporting info item [file IJPO-12-102-s001.zip › sup figures 1.tiff]

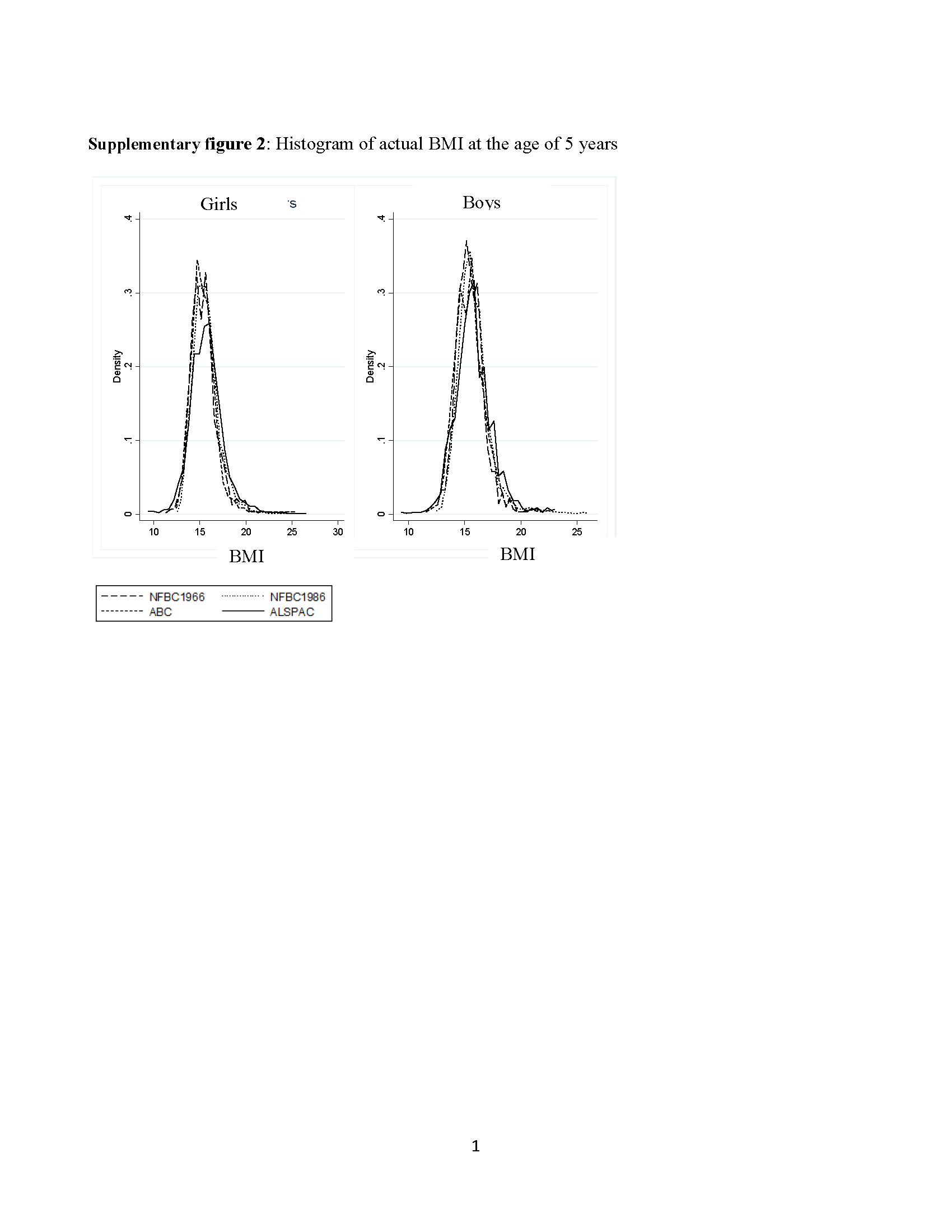

Supplement: Supplementary file 1 — Supporting info item [file IJPO-12-102-s001.zip › sup figures 2.tiff]

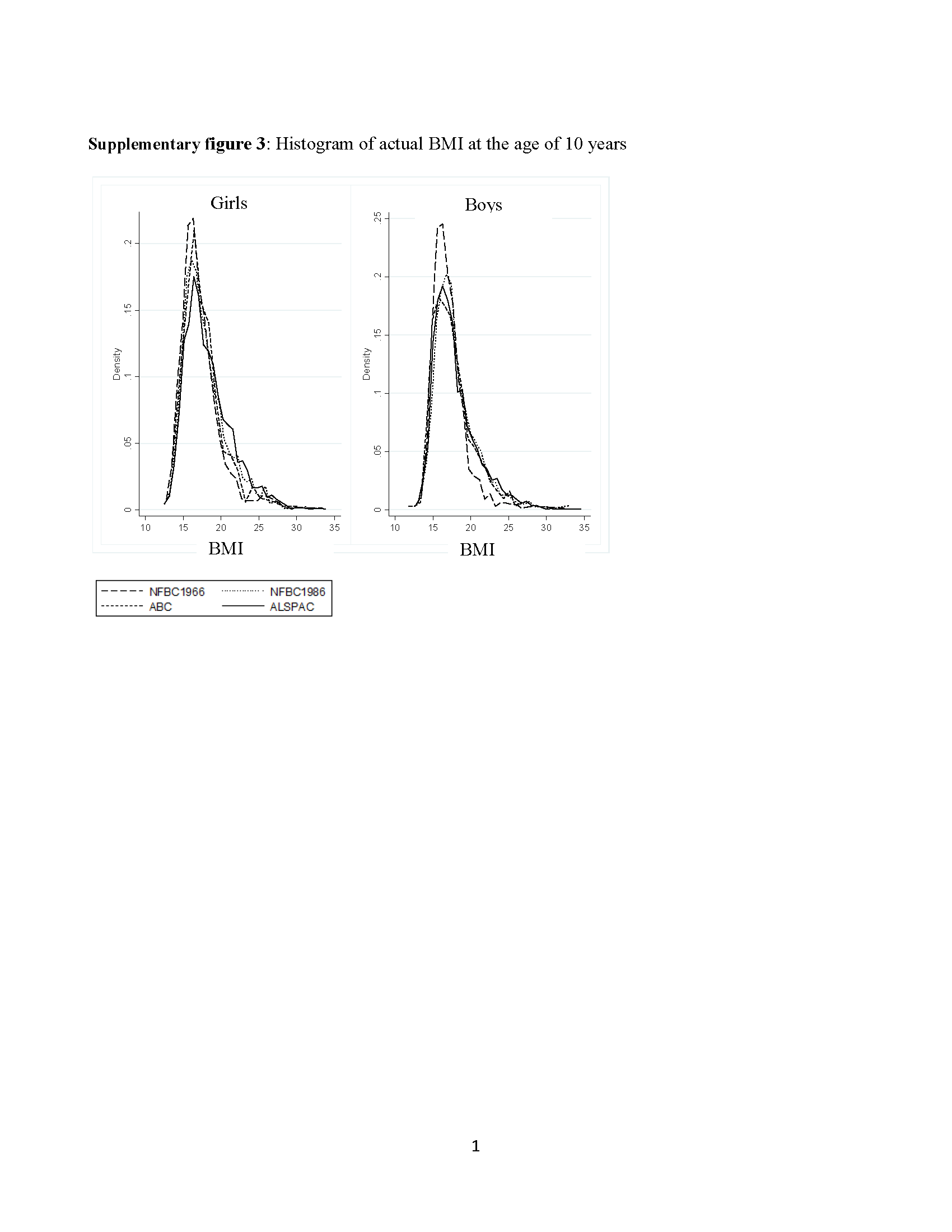

Supplement: Supplementary file 1 — Supporting info item [file IJPO-12-102-s001.zip › sup figures 3.tiff]

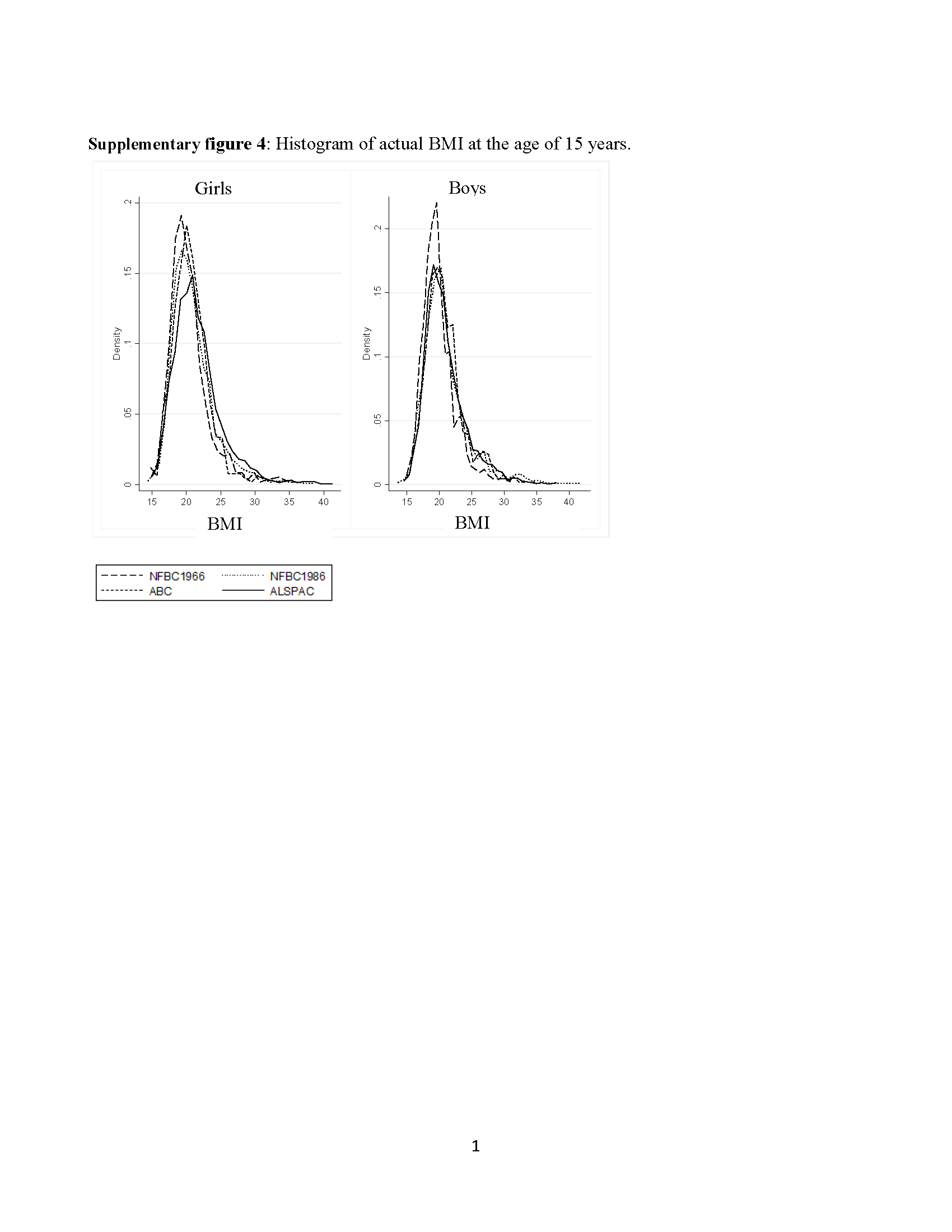

Supplement: Supplementary file 1 — Supporting info item [file IJPO-12-102-s001.zip › sup figures 4.tiff]
